# Golgi phosphoprotein 2 in physiology and in diseases

**DOI:** 10.1186/2045-3701-2-31

**Published:** 2012-09-10

**Authors:** Ha-Jeong Kim, Dandan Lv, Yan Zhang, Tao Peng, Xiaojing Ma

**Affiliations:** 1 Department of Microbiology and Immunology, 1300 York Avenue, New York, NY, 10065, USA; 2 Department of Pediatrics, Weill Cornell Medical College, 1300 York Avenue, New York, NY, 10065, USA; 3 Graduate Program in Immunology and Microbial Pathogenesis, Weill Graduate School of Medical Sciences, Cornell University, 1300 York Avenue, New York, NY, 10065, USA; 4 School of Life Science and Biotechnology, Shanghai Jiao Tong University, Shanghai, 200240, China; 5 State Key Laboratory of Respiratory Disease, Guangzhou Institutes of Biomedicine and Health, Chinese Academy of Sciences, Guangzhou, China

**Keywords:** GOLPH2, Hepatocellular carcinoma, Endosomal trafficking, Viral infection, Cell mediated immunity

## Abstract

Golgi phosphoprotein 2 (GOLPH2, also termed GP73 and GOLM1) is a type II transmembrane protein residing in the cis and medial-Golgi cisternae. GOLPH2 is predominantly expressed in the epithelial cells of many human tissues. Under poorly defined circumstances, GOLPH2 can be cleaved and released to the extracellular space. Despite of its relatively “young age” since the first description in 2000, the physiological and pathological roles of GOLPH2 have been the subject that has attracted considerable amount of attention in recent years. Here, we review the history of GOLPH2’s discovery and the multitude of studies by many groups around the world aimed at understanding its molecular, cellular, physiological, and pathogenic activities in various settings.

## The GOLPH2 gene

The 73 kDa protein is coded by the gene *GOLM1* located on human chromosome 9q21.33 (mouse chromosome 13) and was originally cloned by differential screening of a cDNA library derived from liver tissue of a patient with adult giant-cell hepatitis [1], a rare form of hepatitis with suspected viral etiology. GOLPH2 was also independently identified in “the secreted protein discovery initiative (SPDI), a large-scale effort to identify novel human secreted and transmembrane proteins using a biological signal sequence trap in yeast cells aided by computational tools [2]. The gene is conserved in chimpanzee, dog, cow, mouse, chicken, and zebrafish. The closest human homologue to GOLPH2 is the cancer susceptibility candidate gene 4 (CASC4) protein (Swiss-Prot Q6P4E1), a single-pass type II membrane protein that co-localizes with GOLPH2 (unpublished data of the authors), the increased expression level of which is associated with HER-2/neu proto-oncogene overexpression [3].

GOLPH2 genomic sequence predicts 11 exons and two splicing variants. The transcript variant 1 (NM_016548.3) is 3100nt in length and contains exons 2 to 11, while transcript variant 2 (NM_177937.2) is 3092nt in length and contains exons 1, and 3 to 11. Both variants encode the same open reading frame. The biological significance of these variants is not clear.

## Molecular and biochemical characteristics of GOLPH2

**Figure 1:**
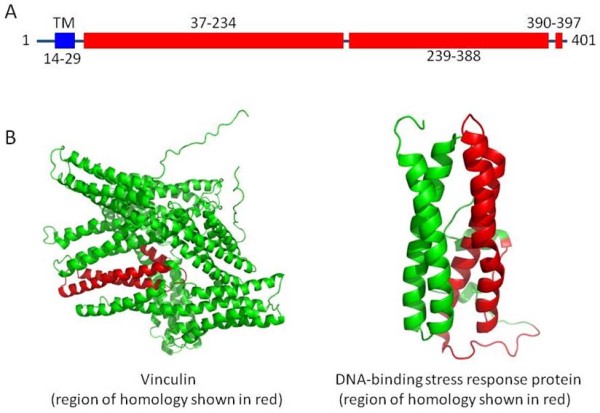
**Structural features of GOLPH2.A**. Predicted transmembrane (TM) and helical regions of the protein. Approximate residues ranges are labeled. **B**. Low but significant sequence homology to proteins with known structures.

Sequence analysis reveals that GOLPH2 contains a predicted transmembrane domain (TMD) at the N-terminal region, consistent with the observation that the protein can be found in the serum or cell culture supernatant, likely by secretion or by a shedding mechanism. Strikingly, it appears that the protein is entirely helical after the TMD, with two predicted continuous helical regions of 150 to 200 residues in length (Figure 1A). This striking helical nature of the protein may explain its observed resistance to proteases (unpublished data of the authors), because proteolysis requires a stretch of extended conformation such as β-strand conformation or random coil conformation. The apparent simplicity of the protein with regards to its secondary structure may also explain the heat resistance of the protein (unpublished data of the authors) because the protein may have an extraordinarily high denaturation temperature or may re-fold readily upon cooling. A search for sequence homology also revealed that a central region of the protein around residues 80–190 share low sequence homology (20-30% identity) to several highly helical proteins, including Vinculin (PDBID 1ST6) and DNA-binding stress response protein (2C2F) (Figure 1B).

The amino acid residues 70–210 may contain a GC-rich sequence DNA-binding factor-like protein domain although the significance of this observation is not clear. Further biochemical and structural characterization of GOLPH2 will help reveal its oligomerization state, stability and structural organization of the helices.

To understand how *GOLPH2* gene expression is regulated, a 2,599-bp human *GOLPH2* promoter fragment was cloned and characterized in epithelial cells including Hela, the HCC-derived HepG2, and breast cancer cell MCF7 [4]. Sequence analysis indicates that GOLPH2 core promoter does not contain the canonical TATA element. Deletion analyses revealed three important domains: a repressive region, a positive regulatory region and a core promoter region. Furthermore, adenoviral early region 1A (E1A) was able to activate the *GOLPH2* promoter, consistent with the original description of GOLPH2’s induction by viral infection [5]. A GC-box motif located at −89 to −83 in the core *GOLPH2* promoter region partly mediated E1A transactivation [4].

## Trafficking of GOLPH2

Under steady-state conditions GOLPH2 is an integral membrane protein of the *cis* and medial-Golgi. However, similarly to the structurally related protein GPP130, it cycles out of the *cis* Golgi to endosomes and the cell surface [6]. There is evidence that the endosomal trafficking of GOLPH2 allows for proprotein convertase furin-mediated cleavage, resulting in its release into the extracellular space, and provides a molecular explanation for its presence as a serum biomarker for HCC [7]. The structural determinants for Golgi localization were investigated using a panel of GOLPH2 truncation mutants [8]. The Golgi localization of GOLPH2 was not affected by the deletion of the C-terminal part of the protein. A truncated mutant containing the N-terminal portion (the cytoplasmic tail and TMD) localized to the Golgi. Sequential deletion analysis of the N-terminus indicated that the TMD with a positively charged residue in the cytoplasmic N-terminal tail were sufficient to support Golgi localization. It was also shown that both endogenous and secreted GOLPH2 exist as a disulfide-bonded dimer, and the coiled-coil domain was sufficient for dimerization [8].

To note, it has been speculated from the beginning that GOLPH2 might be important for protein transportation; however, no direct evidence has been reported yet demonstrating its role in this process.

## GOLPH2 in development

GOLPH2’s physiological importance was inferred by an early study showing that transgenic mice expressing a C-terminally truncated GOLPH2 exhibit decreased survival and hepato-renal pathology with strong inflammatory cell infiltrates [9]. This renal pathology is partially mimicked in the knockout mice for the lipoprotein clusterin (CLU) [10], the secretory form of which (sCLU) has been shown by us to interact with secretory GOLPH2 through the latter’s C-terminus [11].

GOLPH2 is highly conserved among vertebrates, suggesting that it performs a conserved biological function. Indeed, studies revealed impressive levels of similarities between human and Xenopus GOLPH2s. Both proteins are localized in *golgi* and forms dimer in a similar manner. More important, both proteins are highly expressed in epithelial cells, suggesting mechanisms of expression regulation. In an effort to understand the physiological function of GOLPH2, the developmental role of GOLPH2 in the *Xenopus* model was characterized. *Xenopus golph2* is expressed in the pronephros during early development. The morpholino-mediated knockdown of golph2 results in edema formation. Additionally, *Nephrin* expression is enhanced in the glomus, and the expression of pronephric marker genes, such as *atp1b1, ClC-K, NKCC2,* and *NBC1*, is diminished in the tubules and duct. The expression of *WT1* is increased in the glomus and expanded laterally in the pronephric region, which may promote glomus formation and inhibit pronephric tubule differentiation [12]. Continued utilization of a relatively simple but well-defined model system such as *Xenopus laevis* will lead to a better understanding of the biological functions, pathogenesis and other important physiological properties of GOLPH2*.*

## GOLPH2 in liver diseases

Clinical studies first uncovered high levels of GOLPH2 in the sera of patients with liver disease, particularly hepatocellular carcinoma (HCC) [13-15]. Compared with α-fetoprotein (AFP), the most commonly used serum marker for over four decades, GOLPH2 serum levels appear to be more sensitive for early HCC [14,16]. Further, GOLPH2 is hyperfucosylated in HCC, and its hyperfucosylated fraction in serum is an even better disease marker [13]. The most profound elevation of serum levels of GOLPH2 has been detected in patients who have developed HCC on the background of infections by HCV genotype 1b and HBV [17]. A recent Chinese study was conducted with systematic review of related studies, sensitivity, specificity and other measures about the accuracy of serum GOLPH2 and AFP in the diagnosis of HCC using random-effects models. The results of this study indicate that serum GOLPH2 has a comparable accuracy to AFP for the diagnosis of HCC, while the value of serum GOLPH2 in combination with AFP for HCC detection warrants further investigations [18].

In a study aimed to investigate the expression of GOLPH2 and its correlation with clinical parameters, significant overexpression of GOLPH2 at either protein- or mRNA-levels or both were fund to be associated with aggressive behavior of HCC, but not overall patient survival [19]. This observation suggests that GOLPH2 is not merely a marker of HCC progression but it may have HCC-promoting activities.

## GOLPH2 in other diseases

GOLPH2 has been described as an excellent ancillary tissue biomarker for the diagnosis of prostate cancer [20-22]. One study revealed significantly elevated GOLPH2 expression in lung adenocarcinoma tissues [23]. Interestingly, the levels of soluble GOLPH2 (sGOLPH2) were about 30% higher in lung cancer patients compared with healthy individuals [23]. GOLPH2 is consistently overexpressed in seminomas compared with matching non-neoplastic tissues with statistically high significance [24]. GOLPH2 is also highly expressed in the intertubular Leydig cells as well as in Leydig cell tumors [24]. High GOLPH2 expression has been observed in normal renal tubules and in almost half of renal cell carcinomas (RCC) with a statistically significant predominance in the papillary and chromophobe histological subtypes [25].

A case–control Genome-wide association study (GWAS) with replication in Canada and the United Kingdom shows that unadjusted, single nucleotide polymorphism (SNP) rs4420638 within *APOC1* is strongly associated with Alzheimer’s disease (AD) due entirely to linkage disequilibrium with *APOE*. By multivariable adjusted analyses, 3 SNPs within the top 120 by *P* value in the logistic analysis and 1 in the Cox analysis of the Canadian data set provides additional evidence for association at *P*<0.05 within the United Kingdom Medical Research Council data set: rs7019241 (*GOLPH2*), rs10868366 (*GOLPH2*), rs9886784 (chromosome 9), and rs10519262 (intergenic between *ATP8B4* and *SLC27A2*) [26]. In a study of the Han Chinese population, rs10868366 and rs7019241 in GOLPH2 were found to be in strong linkage disequilibrium. Furthermore, the rs10868366 T/rs7019241 T alleles form a relative protective factor whereas the rs10868366 G/rs7019241 C alleles constitute a relative risk factor [27]. Another Chinese study found that the ApoEϵ4-associated risk of AD increased approximately two-fold if the GOLPH2-T allele of rs7019241 was also present, suggesting that GOLPH2 modifies the ApoEϵ4-associated risk of AD [28].

## GOLPH2’s potential activities in the immune system

One of the traditional immunological paradigms is that B-cell and T-cell interactions are a one-way phenomenon of T-cell help to induce the terminal differentiation of B cells to produce antibodies [29,30]. However, recently emerging evidence indicates that B cells may have strong regulatory effects on T cell-mediated immune responses [31-33]. The mechanisms are not well understood. A proteomics-based approach was adopted to identify protein molecules potentially important for this much underexplored but important pathway of immunoregulation (unpublished data of the authors). Initial screening identified a secreted activity by pathogen-activated primary human and mouse B lymphocytes, and by many types of neoplastic B cells spontaneously. This novel activity strongly suppresses T cell responses indirectly by modulating dendritic cell (DC) properties through a selective inhibition of the production of Interleukin-12 (IL-12), a heterodimeric cytokine essential for T_H_1-mediated immunity against intracellular infection and malignant growth. By a combination of biochemical and mass spectrometric analyses, it was further determined that the major activity of this factor was in fact GOLPH2. The “co-incidental” convergence of the autonomously carried out immunological investigations by us and clinical studies by others on GOLPH2 strongly hints at the potential importance of this intriguing molecule. It is tempting to speculate that the sGOLPH2 by HCC may have a pathological role, which is to inhibit IL-12 production by DCs, thwarting T cell responses against malignant development. GOLPH2 may also have immune-independent activities on HCC growth and/or metastasis *in vivo*.

The T_H_1/T_H_2 balance is impaired in many disorders, including infectious and autoimmune diseases and malignancies. In AIDS, for example, there is strong evidence that as the disease progresses hyperactivation of B lymphocytes and cytokine dysregulation occur, and the overproduction of type-2 cytokines (IL-4, IL-13, etc.) promotes T_H_2 immune responses associated with a permissive environment for the survival and persistence of the virus, accompanied by impaired production of type-1 cytokines (IL-12, IFN-γ) and diminished cell mediated immunity (CMI), which is an indispensable and critical mechanism to eliminate HIV infected cells and control the spread of infection [34]. Consistent with these observations, *in vivo* treatment with recombinant IL-12 protects mice from immune abnormalities observed during murine acquired immunodeficiency syndrome (MAIDS) [35]. IL-12 also enhanced the cellular immune response *in vivo* against human HIV-1 env antigen in a DNA prime/vaccinia virus boost vaccine regimen [36].

It is therefore further postulated that hyperactive B cells inhibit IL-12 gene expression in DCs by producing the extracellular GOLPH2, thereby dampening DC/IL-12-driven CMI against persistent and chronic intracellular infections in which B cell activation is dysregulated, such as in HIV/AIDS.

## Conclusions

Given its strong association with a number of important diseases, GOLPH2 is an intriguing molecule that deserves fruther exploration. Future efforts towards understanding its roles and mechanisms in physiological and pathogenic settings can be direted but not limited to the following aspects: (1) to delineate its potential role in mammalian development and cellular differentiation; (2) to further study its role in viral infections of epithelial cells; (3) to differentiate the roles of the intracellular vs the extracellular GOLPH2; (4) to identify its putative cellular receptor through which the sGOLPH2 induces intracellular signaling; (5) to investigate the immunological and non-immune roles of sGOLPH2 in HCC growth and metastasis in immunocompetent and xenographic mouse models of HCC, respectively; (6) to investigate the molecular mechanism of B cell-mediated evasion of CMI via inhibition of IL-12 synthesis in DCs by sGOLPH2.

## Competing interests

The author(s) declare that they have no competing interests.

## Authors’ contributions

HK identified GOLPH2 from B lymphocytes and characterized many of its in vitro activities in macrophages and dendritic cells. DL carried out the IL-12 p35 reporter assays. YZ performed some of the GOLPH2 expression-knockdown studies.TP did most of the biochemical characterization of GOLPH2 as well as the developmental work. XM was responsible for the overall direction of the project and for the writing of this manuscript. All authors read and approved the final manuscript.
